# Endoscopic repair of spontaneous esophageal rupture during gastroscopy: A CARE compliant case report

**DOI:** 10.1097/MD.0000000000013422

**Published:** 2018-11-30

**Authors:** Feiyun He, Mugen Dai, Jiwang Zhou, Jiansheng He, Bin Ye

**Affiliations:** a Department of Gastroenterology, The Fifth Affiliated Hospital of Wenzhou Medical University; b Department of Gastroenterology, Lishui Chinese medicine hospital; c Department of Anorectal surgery, Lishui Chinese medicine hospital, Lishui, Zhejiang Province, China.

**Keywords:** gastroscopy, spontaneous esophageal rupture, Treament

## Abstract

**Rationale:**

Most of esophageal rupture is a very serious life-threatening benign gastrointestinal tract disease with high mortality. However, there are a few cases of spontaneous esophageal rupture during gastroscopy.

**Patient concerns:**

A 57-year-old man who underwent a routine diagnostic gastroscopy due to food obstruction was reported. During the gastroscopy, he vomited severely, which was followed by severe left chest pain radiating into the back and upper abdomen. The diagnosis was made by computed tomography (CT) scan without delay. Enhanced CT showed extensive mediastinal emphysema, a small amount of left pleural effusion, and a 6 cm tear was confirmed in the lower esophagus posteriorly.

**Diagnoses:**

The patient was diagnosed with an intrathoracic rupture type of spontaneous esophageal rupture.

**Interventions:**

The patient received endoscopic suturing techniques under endotracheal intubation, titanium clip clamping, and over the scope clip (OTSC) sealing.

**Outcomes:**

The procedure was smooth and the patient recovered well after operation.

**Lessons:**

During gastroscopy, the risk of esophageal rupture should be considered due to sudden pain caused by severe nausea and vomiting. Esophageal rupture can rapidly lead to severe life-threatening infections such as empyema and mediastinitis. Therefore, awareness of this condition is important so that appropriate treatment can rapidly be implemented to increase the likelihood of a good outcome.

## 1 Introduction

Diagnostic gastrointestinal tract endoscopy is well established as a safe procedure. During gastroscopy, little is known about the potential danger of accidental spontaneous rupture of the esophagus, and life is often at risk. Spontaneous rupture of esophagus is a kind of rare rupture of the wall of the esophagus, which is caused by non-traumatic rupture of the total thickness of the wall of the esophagus.

The incidence rate is 1:6000, but the mortality rate is as high as 25% to 100%.^[1–4]^ The onset of the disease is sudden and serious. If not treated in time, acute vertical sputum inflammation, esophageal pleural palsy, can lead to death.^[5]^ Due to the low incidence rate, atypical medical history, and high misdiagnosis rate (74.3%),^[6]^ it is easy to delay the timing of surgery. A rare case of spontaneous rupture of esophagus under gastroscope is reported. The diagnosis is accurate and the endoscopic suture technique is superior to the traditional operation.

## 2 Case presentation

**Figure 1:**
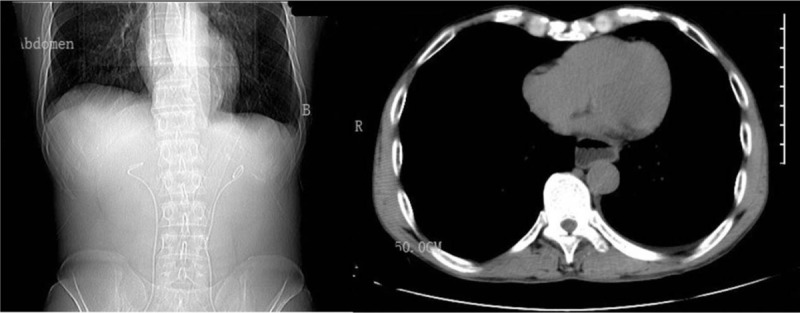
The esophagus computed tomography scan is normal before gastroscopy.

A 57-year-old man underwent gastroscopy because of eating obstruction. He had history of surgery for gastric cancer. The esophagus computed tomography (CT) scan (Fig. 1) was normal before gastroscopy. Laboratory analysis revealed the following: serum RBC 4.7cell/L; Hgb 138 g/L; HCT 42.8% when just hospitalized. On the 6th day after admission, the endoscopic jejunal tube placement was performed under the gastroscope, and parenteral nutrition was performed. There was no discomfort after operation. After 25 days of operation, the nutrient tube was blocked and removed. It is planned to further perform gastroscopy jejunal tube placement.

**Figure 2:**
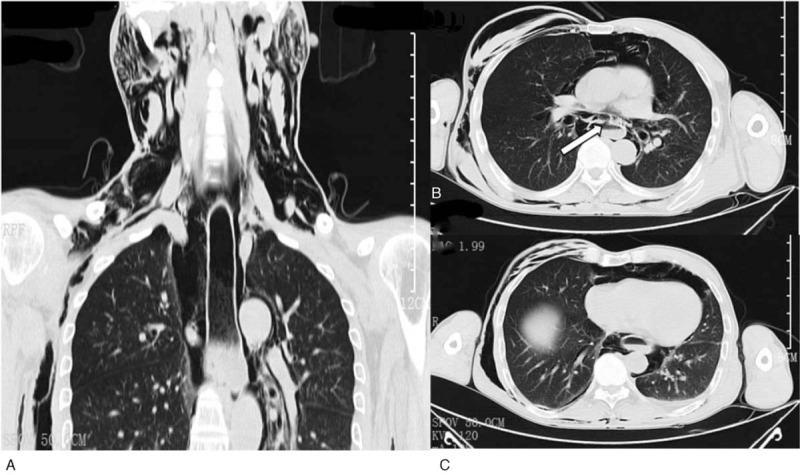
CT scan of the chest was showed extensive subcutaneous emphysema in the chest and back, a large amount of emphysema in the mediastinum, a small amount of left pleural effusion. (B arrowhead, Esophageal rupture). CT = computed tomography.

During the gastroscopy process, the patient suddenly suffered from nausea and vomiting, felt severe pain in the chest and lower back, and felt a compression pain in the neck. A physical examination revealed subcutaneous emphysema in the thoracolumbar segment and face and neck, with a crepitus. An emergency CT scan of the chest and neck showed extensive subcutaneous emphysema in the chest and back, a large amount of emphysema in the mediastinum, a small amount of left pleural effusion, no pneumothorax (Fig. 2). Spontaneous esophageal rupture was diagnosed. Laboratory analysis revealed that serum RBC 3.3cell/L; Hgb 100 g/L; HCT 28.5% after spontaneous esophageal rupture.

**Figure 3:**
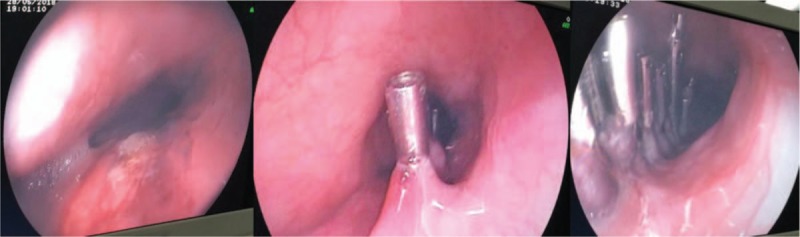
Endoscopic titanium clip clamping esophageal chasm under endotracheal intubation.

**Figure 4:**
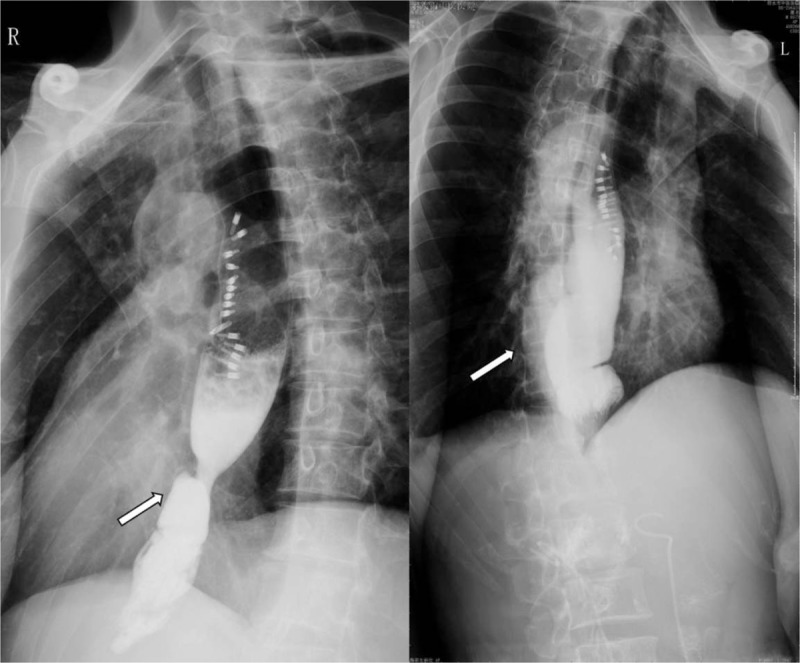
X-ray scan of the chest, X-ray contrast study of the esophagus. The arrow indicates the esophagus without the tracer leakage. (As arrowhead was shown).

Due to the poor general condition of the patient, the risk of surgery was high, and endoscopic treatment was preferred. A long strip rupture in esophagus which was 28–34 cm away from the incisors was seen by endoscope. A small amount of blood stasis was applied, and a hemostatic clip was placed from the anal side to the mouth side and a large Boston's clip was stitched. A total of 14 titanium clips were used for suturing the split (Fig. 3). Postoperative fasting and strong anti-infection treatment were performed. Esophageal angiography was performed on the 11th day after operation. No contrast agent leakage or exacerbation of emphysema was observed (Fig. 4).

**Figure 5:**
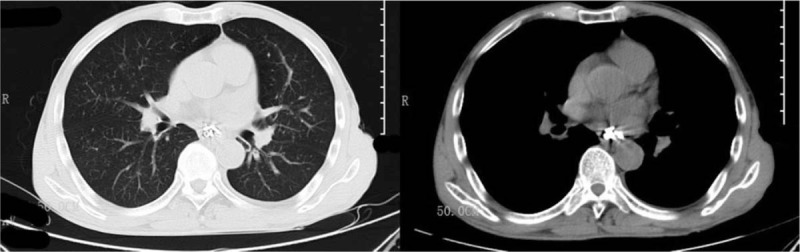
Recheck chest CT was showed mediastinal emphysema and subcutaneous emphysema disappeared 17 days after the operation. CT = computed tomography.

Recheck chest CT was performed after the operation. 17 days later, emphysema and subcutaneous emphysema disappeared (Fig. 5). Laboratory analysis revealed serum RBC 3.98cell/L; Hgb 113 g/L; HCT 33.6% 20 days later after the operation. Recovery was complicated by renal failure, leading to death 61 days after admission.

## 3 Discussion

Spontaneous esophageal rupture, also known as Boerhaave syndrome^[7]^ is a rare but severe gastrointestinal disease, its early symptoms are similar to chest and abdomen emergencies, diagnosis and treatment are often delayed, in some cases will lead to adverse results.^[8]^ The rupture of the esophagus most commonly results from a full-thickness transmural rupture of the normal esophageal wall due to a sudden increase in intraesophageal pressure caused by nausea or vomiting.^[9]^ Spontaneous esophageal rupture has been reported, including patients with gastrointestinal stenosis, Barrett's esophagus, vomiting during pre-treatment for an endoscopic examination, and vomiting after general anesthesia.^[10]^ In most cases of spontaneous esophageal rupture, the tearing along the fiber is mostly longitudinal slit, generally 0.6–8.9 cm, can also reach 10–12 cm, 90% occurs in the left chest, mostly in the lower left of the esophagus, and also into the right side and the abdominal cavity.^[11]^ Most of the clinical manifestations are atypical. Early manifestations include sudden chest pain or upper abdominal pain, radiation to the shoulders and back, accompanied by difficulty in breathing, chest tightness, fever and other symptoms.^[12]^ This characteristic history is the key to diagnosis of spontaneous rupture of esophagus and should be regarded as the first possible diagnosis. Chest radiographs and CT are important in the diagnosis of subcutaneous emphysema, mediastinal emphysema and pleural effusion.

Gastroscopy is a remarkably safe and effective method of diagnosing gastrointestinal tract disease, widely used in clinical setup. Most of the complications can be avoided by complying with general gastroscopy rules. Due to its rare incidence, most spontaneous perforation of the esophagus during diagnostic gastroscopy reports in the medical literature are case reports. Only 2 similar cases of this article reported previously. The 2 patients were both elderly, one was a 1.5 cm tear was confirmed in the lower esophagus posteriorly,^[13]^ other was a 5 cm tear in the left lateral wall of the esophagus.^[14]^ Our patient was a 6 cm tear in the lower esophagus posteriorly. Since the establishment of our hospital, this is the first patient of spontaneous esophageal rupture induced by gastroscopy, even domestic. This patient mainly due to the following reasons: Nausea and vomiting were evident during gastroscopy, leading to a significant increase in hypoesophageal pressure. In addition, the patient had poor nutritional status, thin lower esophagus muscle layer, leading to spontaneous esophageal rupture.

Esophageal rupture is the most serious and rapidly lethal perforation of the gastrointestinal tract, once diagnosed, immediate treatment was needed.^[15]^ Delayed diagnosis and treatment can rapidly lead to severe life-threatening infections such as empyema and mediastinitis, and multiple organ failure. The treatment strives to be carried out within 24 h of diagnosis, and the mortality rate of patients exceeding 24 h is extremely high. The principle of treatment is to remove the source of pollution, close the breach, restore the integrity of the esophagus, fully drain, control the infection, strengthen the nutritional support, improve the body and promote wound healing. Traditional surgical treatment is the primary suture repair of the rupture and adequate drainage time of the mediastinum and chest.^[16]^ However, for this patient, the doctor tried endoscopic suturing techniques under endotracheal intubation, performed titanium clip clamping, and over the scope clip (OTSC) sealing.^[17]^ The procedure was smooth and the patient recovered well after operation. This endoscopic repair is less invasive and infective, and is very useful to patients.

In short, spontaneous esophageal rupture is an emergency and requires early diagnosis if death or serious long-term disease is to be avoided. A patient with spontaneous esophageal rupture was encountered during gastrointestinal endoscopy, and the disease was diagnosed by CT and endoscopically treated at an early stage of rupture. and postoperative recovery was satisfactory. For patients with severe malnutrition after gastric cancer surgery, there is a risk of spontaneous esophageal rupture during gastroscopy. Therefore, it is important to recognize this emergency so that appropriate treatment can be tried quickly to increase good results.

## Author contributions

**Conceptualization:** Bin Ye.

**Data curation:** Feiyun He, Mugen Dai, Jiwang Zhou, Jiansheng He.

**Formal analysis:** Bin Ye.

**Funding acquisition:** Bin Ye.

**Investigation:** Feiyun He.

**Methodology:** Mugen Dai.

**Project administration:** Bin Ye.

**Resources:** Bin Ye, Jiwang Zhou, Jiansheng He.

**Supervision:** Jiansheng He.

**Validation:** Jiansheng He.

**Writing – original draft:** Feiyun He, Mugen Dai, Bin Ye.

**Writing – review and editing:** Feiyun He, Mugen Dai, Jiwang Zhou, Jiansheng He, Bin Ye.
